# Occurrence of viral and bacterial pathogens in South American camelids in representative German flocks

**DOI:** 10.3389/fvets.2026.1744335

**Published:** 2026-03-13

**Authors:** Christian Menge, Stefanie A. Barth, Eva-Maria Bartl, Christian Berens, Christa Ewers, Carsten Heydel, Hannah Hümmelchen, Heike Köhler, Patricia König, Alina Leisen, Svenja Mamerow, Falk Melzer, Katja Mertens-Scholz, Ulrich Methner, Aung Zaw Moe, Dennis Rubbenstroth, Christiane Schnee, Christian Seyboldt, Lisa Ulrich, Kerstin Wernike, Hermann Willems, Henrik Wagner

**Affiliations:** 1 Friedrich-Loeffler-Institut, Institute of Molecular Pathogenesis, Jena, Germany; 2 Veterinary Clinic for Reproductive Medicine and Neonatology, Justus Liebig University Giessen, Giessen, Germany; 3 Institute of Hygiene and Infectious Diseases of Animals, Justus Liebig University Giessen, Giessen, Germany; 4 Friedrich-Loeffler-Institut, Institute of Diagnostic Virology, Greifswald-Insel Riems, Germany; 5 Friedrich-Loeffler-Institut, Institute of Bacterial Infections and Zoonoses, Jena, Germany; 6 Clinic for Swine–Herd Medicine and Molecular Diagnostics, Justus Liebig University Giessen, Giessen, Germany

**Keywords:** alpaca, bacterial pathogens, Germany, huarizo, llama, South American camelids, viral pathogens, zoonosis

## Abstract

Rapidly growing populations of South American camelids (SAC), introduced to Europe as non-indigenous species, have increased contacts at the livestock and human interfaces. This study assessed the occurrence of epizootic and zoonotic viral and bacterial pathogens of prime importance on 10 farms, selected to mirror the diversity of German SAC holdings in size and animal use. Farms were visited four times at approximately 6-months intervals, with 20 animals sampled per visit, resulting in 739 blood and 723 fecal samples from 449 animals (292 alpacas, 156 llamas, one huarizo). Wherever possible, diagnostic methods applied followed national or international recommendations. Antibodies against Schmallenberg virus were detected in 54.6% of animals. Only three animals showed reactivity against Borna disease Virus 1 and one against bovine viral diarrhea virus (BVDV) 1. All animals tested negative for BVDV-2, border disease virus and bovine herpesvirus 1. Thirty-six samples from 30 SAC yielded a non-negative, presumably false-positive results for antibodies against the *Mycobacterium tuberculosis* complex. Ten samples from six animals were non-negative in an iELISA for brucellae antibodies, but all tested negative by Complement Fixation Test. *Coxiella burnetii*-specific antibodies were detected in three individuals from two different flocks, and a single fecal sample tested PCR-positive for *C. burnetii*. *Chlamydiaceae*-specific antibodies were found in 9 of 10 flocks and in 13.6% of the animals, with chlamydial fecal shedding observed in 8 of 10 flocks and in 29.6% of the animals. The animal positivity rate for Candidatus *Mycoplasma haemolamae* was 31.6%. *Mycobacterium avium* subsp. *paratuberculosis* was isolated from 0.34% and *C. difficile* from 2.7% of the animals. While *Salmonella* Typhimurium was found in only 1 of 719 fecal samples, thermotolerant *Campylobacter* (16 *C. jejuni* and 7 *C. coli*) were isolated from 4.8% of the animals. The overall rate of *stx*-positive samples, indicative of Shiga toxin-producing *Escherichia coli* (STEC) shedding, was 32.4%. Based on these detection rates, SAC do not appear to pose a greater risk of infection than other German livestock species at present. However, SAC represent a novel reservoir host that could disturb established and monitored indigenous epizootic transmission networks including those of enteric and abortifacient zoonotic agents.

## 1 Introduction

Starting in the 1980s, South American camelids (SAC) have been commercially imported in significant numbers into several regions worldwide, where they are non-indigenous. Current estimates amount to, e.g., 100,000 SAC in Australia and 90,000 in the U.S. (1, 2). They were introduced as farmed animals with increasing popularity to the central European livestock husbandry landscape with, e.g., 4,500 alpacas kept in Ireland (3), about 35,000 alpacas and 4,000 llamas in the UK (4) and an estimated 10,000 llamas and alpacas in Austria (5). The number of SAC in Germany has also risen sharply over the last 10 years (6, 7). Although there is no legal obligation for SAC registration in Germany, breeding associations estimate that their members keep between 30,000 and 35,000 animals (HW, personal communications). A recent survey among 43 private SAC holdings in central Germany showed that most animals are kept in small holdings with less than 20 individuals (75%). SAC were frequently co-housed with other animal species (30 of 41 holdings) and most holdings (73%, *n* = 31/41) regularly welcomed visitors (8).

Parasitic infestations, with both, endo- and ectoparasites, are the most frequently recognized health problem in SAC holdings (6). In Europe, SAC become confronted with the regional endoparasite populations of ruminants and the animals may share several helminths with sheep, goats and cattle, making co-grazing with ruminants a risk factor for the establishment of high endoparasite burdens in camelid herds (9). Conversely, alpacas could also serve as a reservoir for the trichostrongylid *Haemonchus contortus*, potentially contributing to ruminant infections (9). This is of major health concern as high rates of resistance to anthelmintic drugs in strongylids from German alpaca herds have been reported (9, 10). Among zoonotic disease agents identified in samples from SAC in England and Wales that were sent in for disease investigation between 2000 and 2015, tuberculosis, cryptosporidiosis, sarcoptic mange and Shiga toxin-producing *Escherichia coli* (STEC) were repeatedly found (4).

Despite frequent close contact of SAC to livestock and humans, data on the prevalence of epizootic and zoonotic viruses and bacteria in SAC remain sparse. Being susceptible for a number of epizootic agents affecting small and large ruminants as well as agents affecting equids, several field studies have confirmed SAC both as natural and dead-end hosts for infectious agents (11, 12). Even though shedding of viral and bacterial pathogens of public health concern by SAC at livestock-livestock and livestock-human interfaces seems to be relatively uncommon at present, reported positive cases highlight the need for improved surveillance (8). This particularly applies for countries like Germany, where several current trends, i.e., (i) increasing absolute numbers of SAC, leading to more frequent interspecies contacts with other livestock species, (ii) more intensive commercial use of SAC as petting or trekking animals or in animal-assisted therapy (6) resulting in very close SAC-to-human contact, and (iii) the recent emergence of novel vectors and vector-borne diseases in central Europe, coincide with an environment to which the animals are non-indigenous. It is reasonable to suspect that these developments may impact on infectious agents' transmission networks, potentially destabilizing long-established equilibria if they continue and if the epizootic situation is not properly monitored. Of note, camelids, including SAC, have only recently been systematically included in the European legislation on animal health (EU 2016/423). This has resulted in both, a paucity of pre-existing animal health data and an immature portfolio of validated diagnostic tests.

In an attempt to improve our understanding of the current occurrence and distribution of epizootic and zoonotic agents in Germany, and to provide a basis for decision making on future surveillance efforts, this study aimed at conducting a comprehensive multi-pathogen screening of selected SAC flocks in Germany representing the full range of flock sizes and animal uses currently realized in the country. The infectious agents to be addressed were selected based on existing knowledge from individual studies, the German national animal disease reporting system (TSN; https://tsis.fli.de/cadenza/) and the categorization of the diseases according to the European Animal Health Law. To properly appraise the occurrence of selected epizootic and zoonotic pathogens, we (i) assessed the applicability of indirect diagnostic tests established for ruminants to camelids for pathogens for which direct detection was not feasible, and (ii) mitigated the potential impact of intermittent shedding of pathogens on carriage rate estimations by repeated flock sampling.

## 2 Materials and methods

### 2.1 Rationale for the selection of representative flocks

This study capitalized on samples and data collated in the project “Development and establishment of a multi-stage animal health management system for farms with South American camelids.” The project had previously undertaken a survey of livestock farmers, the results of which have been published (7, 13). The survey gathered information on animal holding and welfare conditions, feeding practices, the direction of use and the reproduction scheme realized in the flock as well as the background knowledge of the animal owner. Flocks for the present study were selected from the participants for the survey based on several criteria (see Table 1 for an overview): (1) owners preferably run their SAC husbandry as their main occupation (applied for six out of 10 farms whereas four run their business as a sideline), (2) owners keep high animal numbers (more than 50 SAC), (3) although alpacas are predominantly kept in Germany, llama flocks were also to be included, (4) to collect a nationally representative data set, an effort was made to include flocks from all regions of Germany, and (5) owners utilize their animals for different purposes. The study was conducted in strict accordance with German law for the care and use of experimental animals. Sampling was approved by the competent authority (Regierungspräsidium Giessen, reference number V 54–19 c 20 15 h 02 GI 18/4 Nr. A 3/2021, date of permission: 29.04.2021), and recognized by the competent authorities of the federal states, where the flocks were located. Owners received a cover letter explaining the purpose of the investigation and signed a declaration of consent in accordance with Directive 95/46/EC (General Data Protection Regulation) to allow sampling and analysis for scientific purposes, while ensuring confidentiality.

**Table 1:** Overview of flocks and fecal sample positivity rates for bacterial pathogens.

**Flock**				**Rate of positive samples (%) per sampling round** ^**a**^															
	**Localization (federal state)**	**No of SAC (** ***n*** **) sampled**	**No Alpacas/No llamas (** ***n*** **/** ***n*** **) sampled**	***C. difficile***		***C. perfr***.		***Campylobacter*** **spp**.				***Chlamydia*** **spp**.				**STEC (** ***stx*** **-positive)**			
				**Sampling round** ^b^															
				**A**	**B**	**A**	**B**	**A**	**B**	**C**	**D**	**A**	**B**	**C**	**D**	**A**	**B**	**C**	**D**
1	Hesse	67	33/34	0.0	0.0	10.0	10.0	0.0	0.0	0.0	0.0	50.0	30.0	10.5	80.0	10.0	45.0	20.0	15.0
2	Hesse	24	5/19	0.0	0.0	5.0	0.0	0.0	0.0	5.3	0.0	10.0	5.6	0.0	57.9	0.0	5.0	10.0	20.0
3	Saarland	50	28/22	0.0	5.0	0.0	5.0	0.0	0.0	0.0	0.0	0.0	5.0	0.0	0.0	15.0	30.0	15.0	20.0
4	Bavaria	56	56/0	10.0	10.0	40.0	20.0	20.0	10.0	30.0	10.0	25.0	55.0	10.0	26.3	40.0	35.0	35.0	40.0
5	Bavaria	45	0/45	0.0	0.0	18.8	0.0	0.0	0.0	10.5	0.0	0.0	0.0	0.0	0.0	35.0	45.0	40.0	45.0
6	North Rhine-Westphalia	42	42/0	0.0	10.0	5.0	0.0	0.0	5.0	0.0	6.7	10.0	15.0	15.8	5.3	5.0	25.0	30.0	20.0
7	North Rhine-Westphalia	68	68/0	0.0	0.0	10.5	0.0	5.3	0.0	0.0	0.0	36.8	5.0	30.0	21.1	10.0	60.0	95.0	55.0
8	Rhineland-Palatinate	38^*^	1/36^*^	0.0	0.0	15.8	15.0	0.0	0.0	0.0	0.0	5.3	80.0	5.0	40.0	50.0	35.0	50.0	8.0
9	Saxony	20	20/0	0.0		0.0		10.5				0.0				10.0			
10	Saxony	39	39/0	0.0	5.0	5.0	10.0	5.0	0.0	0.0	0.0	20.0	35.0	20.0	35.0	40.0	35.0	25.0	10.0
Total		449^*^	292/156																

SAC, South American camelids.

^*^Including 1 huarizo.

^a^Empty fields: no samples available; percentage of positive samples independent whether the animals have also been tested in other sampling rounds; numbers indicate the sample positivity rate among all samples suitable for testing as not all of the samples taken per round (n = 20) were available; *C. perfr., Clostridium perfringens*.

^b^Sampling rounds (month/year): A = 04/2021−09/2021, B = 11/2021−04/2022, C = 05/2022−09/2022, D = 11/2022−02/2023.

### 2.2 Sampling

The farms were sampled four times (sampling rounds A—D) at intervals of approximately 6 months (Table 1). A number of 20 animals per farm visit was chosen for practical and compliance reasons. Depending on the size of the farm, several animals were sampled repeatedly. The animals were required to be at least 2 years old, selected and classified as “healthy” by the animal owner prior to the study. For each animal, age, breed, sex, origin, and color were documented. All animals underwent a general clinical examination. The state of care and the body condition score were assessed, and the color of the mucous membranes was documented. In addition, clinical abnormalities such as skin lesions or limb deformities were recorded. Because of material shortages and logistical constraints not all samples were subjected to all diagnostic tests. The applicable numbers are provided in Section 3.

Fecal samples (swabs and solid fecal matter) were taken directly from the rectum. Approximately 25 g of fecal matter, collected by a glove-protected hand, was put in 100 ml containers thereby avoiding cross-contamination between samples and animals. For blood-based diagnostic tests, 5 ml of blood was collected from the left jugular vein in EDTA-containing tubes (KABE EDTA K3E; KABE-Labortechnik GmbH, Nümbrecht, Germany) and 15 ml in tubes without anticoagulant (KABE Serum CAT). From the latter, serum was harvested by centrifugation (2,808 × *g*, 5 min) the following day, cooled to 4 °C and sent together with the fecal samples by express delivery to the distributing laboratory at FLI (Jena) or personally handed over to collaborating laboratories within a walking distance (Giessen).

### 2.3 Preanalytical handling of samples

Rectal swabs for *Campylobacter* cultural detection were transported to the Institute of Hygiene and Infectious Diseases of Animals, Giessen, in Amies transport medium supplemented with charcoal (Nerbe Plus GmbH & Co. KG, Winsen/Luhe, Germany) and freshly streaked onto agar plates (see below).

Upon arrival of the fecal matter samples at FLI Jena, they were immediately subdivided and distributed to the individual laboratories on site. Samples were directly processed for DNA extraction (molecular genetic detection of *Chlamydia* spp. and *Coxiella* sp.) and for the cultural detection of *Salmonella* spp. Other aliquots were kept frozen at−20 °C until the cultural detection of *Clostridium* spp. and *M. avium* subsp. *paratuberculosis*, and at −80 °C until cultural detection of STEC was initiated. Serum samples were also immediately subdivided at FLI Jena, distributed to the respective laboratories at FLI in Jena and Greifswald—Isle of Riems, and stored at −20 °C until further analysis.

### 2.4 Serological testing for viral pathogens

The sera were tested for the presence of antibodies against ruminant pestiviruses [bovine viral diarrhea virus (BVDV), border disease virus (BDV)], Schmallenberg virus (SBV) and bovine herpesvirus 1 (BoHV-1 or BHV-1) by using the commercially available ELISAs ID Screen BVD p80 Antibody Competition, ID Screen^®^ Schmallenberg virus Competition Multi-species (both Innovative Diagnostics, Grabels, France), and the blocking test “Cattletype BHV1 gB Ab” (Indical Bioscience, Leipzig, Germany), all performed according to the manufacturers' instructions. Samples that reacted seropositive for ruminant pestiviruses were subsequently analyzed by microneutralization tests against the BVDV-1 strain “NADL,” the BVDV-2 strain “CS8644” and the BDV strain “Gifhorn,” following the procedures described in the German official collection of test methods for bovine viral diarrhea (14).

To test for seroreactivity against Borna disease virus 1 (BoDV-1), a two-step screening approach using two different variants of an immunofluorescence antibody test (IFAT) was applied following previously published procedures (12, 15). In a first step, all samples were tested using formalin-fixed BoDV-1-infected Vero cells as the antigen source. Samples showing reactivity in this initial screening were then subjected to a confirmatory test using heat-fixed BoDV-1-infected SK6 cells. Only samples showing reactivity in both assays were considered potentially positive for BoDV-1-specific reactivity. Results are reported as end point titration titers.

### 2.5 Serological testing for bacterial pathogens

Sera were tested for tuberculosis-specific antibodies using the INgezim Tuberculosis DR-ELISA (INgenasa, Madrid, Spain; now Gold Standard Diagnostics) diluted 1:25 [v/v] and in duplicates, following the instructions of the European Reference Laboratory for Bovine Tuberculosis (16). According to the positive and negative controls of the assay, each plate was validated and the plate specific cut-offs were calculated (negative value ≤ mean of the negative control + 0.3; positive value > mean of the negative control + 0.35).

*C. burnetii*-specific antibodies were detected using the IDEXX Q Fever (*Coxiella burnetii*) Antibody Test Kit (IDEXX B.V., Hoofddorp, The Netherlands) according to the manufacturer's guidelines, although the kit is not licensed for the use with SAC serum samples. Positive and suspicious samples were re-tested in duplicate. Pre-immune and immune sera from a COXEVAC (Ceva Sante Animale, Libourne, France) vaccinated alpaca served as external negative and positive controls. Vaccination had been carried out as two subcutaneous injections with 1 ml COXEVAC 4 weeks apart. Experimental procedures were approved by the competent authority of the State of Mecklenburg-Western Pomerania, Germany (permit no 7221.3-2-042/17, date of permission: 12.02.2018).

Antibodies against *Chlamydia* spp. were detected using the IDEXX ELISA Chlamydiosis Total Ab Test (IDEXX). All IDEXX *Chlamydia*-positive sera along with three randomly selected negative sera per flock and sampling round were further tested for the presence of *Chlamydia abortus*-specific antibodies using the ELISA ID Screen^®^ *Chlamydophila abortus* Indirect Multi-species (Innovative Diagnostics). Neither test had been validated for use with camelid sera and no defined positive sera were available to establish cut-off values with these samples. Therefore, protocols and cut-offs provided by the manufacturers for ruminant sera were used.

Serum samples were tested for antibodies against *Brucella* (*B*.) *abortus, B. suis* and *B. melitensis* using an indirect ELISA (iELISA) called ID Screen^®^ Brucellosis Serum Indirect Multi-species (Innovative Diagnostics) as specified by the manufacturer. Although the ELISA has not yet been validated for camelids, the specified limit values were used to evaluate the results. Samples yielding a positive or a questionable result were additionally tested using the complement fixation reaction (CFT). The method was carried out as described in the German official collection of test methods for Brucellosis in cattle, pigs, sheep and goats (17). In the absence of camelid-specific validation data, the cut-off values for cattle were used for evaluation.

### 2.6 Screening for *Mycoplasma haemolamae*

EDTA blood samples were stored at 4 °C for up to 48 h until arrival at the laboratory of the Swine Clinic, Justus Liebig University Giessen. DNA was extracted from 150 μl of EDTA blood using the delta PREP INSTANT Virus RNA Kit^®^ (IST Innucreen GmbH, Berlin, Germany), following the manufacturer's instructions. This was followed by amplification of a 16S rRNA gene fragment (309 bp) with the primers CMhlF2 and CMhlR2 and a PCR Mastermix (QIAGEN Multiplex PCR Kit; Qiagen, Hilden, Germany). An internal control resulting in an amplicon of 1,022 bp served as a positive control. PCR products were analyzed by agarose gel electrophoresis. The assay had a specificity of 100% and a sensitivity of < 10 DNA copies per PCR reaction.

### 2.7 Screening for *Chlamydia* spp. and *Coxiella burnetii*

DNA from fecal samples was extracted using the High Pure PCR Template Preparation Kit (Roche Life Science, Penzberg, Germany) according to the manufacturer's protocol with an elution volume of 200 μl.

All samples were initially examined using a 23S-rRNA gene-based *Chlamydiaceae*-specific real-time PCR (qPCR) with a limit of detection of five copies per reaction (18). QuantiTect Multiplex PCR Master Mix (Qiagen) was used in a total volume of 15 μl, containing 300 nM of each primer, 200 nM probe and 2 μl template DNA. PCR inhibition was assessed using an internal amplification control system using primers EGFP-1F and EGFP-10R according to (19), included in duplex qPCR runs. The samples were tested in duplicate on a Bio-Rad CFX96 PCR instrument (Bio-Rad, Feldkirchen, Germany) with the following cycling conditions: 95 °C for 10 min and 45 cycles of 95 °C for 15 s and 60 °C for 1 min. A mean cycle threshold (Cq) value of < 38 was considered positive. 23S-PCR-positive samples were further characterized using species-specific qPCR assays for *Chlamydia* [*C*.] *abortus, C. pecorum, C. psittaci* and *C. suis* (20). Primer concentrations of 800 nM each and probe concentrations of 200 nM were used. Master mix and qPCR cycling conditions were as described above and a mean cycle threshold (Cq) value of < 40 was considered positive.

*C. burnetii-*specific DNA was detected using qPCR targeting the multicopy IS1111 element as previously described with minor modifications (21). Reactions were carried out using the Luna Universal Probe qPCR Master Mix (New England Biolabs GmbH, Frankfurt am Main, Germany) in a total volume of 20 μl, containing 800 nM of each primer, 300 nM probe and 2 μl template DNA. A serially diluted synthesized IS1111 gene fragment (500 bp, Eurofins Genomics Europe Shared Services GmbH, Ebersberg, Germany) was used as a standard. Samples were tested once under the following cycling conditions; 95 °C for 10 min followed by 45 cycles of 95 °C for 15 s and 60 °C for 30 s. To test for inhibition, all negative samples were spiked with 10^3^ IS1111 copies/reaction and re-analyzed. The limit of detection (LOD_95_) for this assay was determined as 20.58 IS1111 copies per reaction (95% confidence interval 15.67; 27.04; data not shown). Results were evaluated according to a Cq value of ≤ 35 as positive, a Cq value > 35 to < 37 as suspicious and > 37 as negative.

### 2.8 Screening for *Mycobacterium avium* subsp. *paratuberculosis* (MAP)

Fecal samples were examined by bacterial culture according to the German official collection of test methods for *Mycobacterium avium* subsp. *paratuberculosis* (22). Briefly, 3 g of feces were decontaminated for 48 h at room temperature using 30 ml of 0.75% hexadecylpyridinium chloride monohydrate solution (Sigma Aldrich, Taufkirchen, Germany). The supernatants were discarded and 200 μl of the remainder were transferred on each of two slopes of Herrold's Egg Yolk Agar supplemented with Mycobactin J plus Amphotericin B, Nalidixic acid and Vancomycin (HEYM, Becton Dickinson, Sparks, NV, USA) and in two slopes of modified Middlebrook 7H9 Medium containing Albumin, Dextrose and Katalase, Mycobactin J, Egg Yolk and Polymyxin B, Amphotericin B, Nalidixic acid, Trimethoprim and Azlocillin (M7H9C) (23). HEYM cultures were incubated at 37 °C for up to 6 months and examined every second week for contamination and bacterial growth. M7H9C cultures were incubated at 37 °C for 3 months. Then, 500 μl of culture medium were removed. The presence of MAP was confirmed in characteristic colonies from HEYM slopes and in M7H9C medium by IS900 PCR (24). Samples showing contamination were considered not analyzable.

### 2.9 Screening for *Clostridioides difficile* and *Clostridium perfringens*

Isolation and characterization of *Clostridioides* (*C*.) *difficile* involved both direct plating and pre-enrichment as previously described (25). Briefly, fecal samples were processed using *C. difficile* moxalactam/norfloxacin agar plates (*Clostridium difficile* Agar Base, CDMN selective supplement, Oxoid, Microbiology Thermo Fisher Scientific, Wesel, Germany) and ChromID *C. difficile* agar (bioMérieux, Nürtingen, Germany) for direct plating, and TCDMN broth (CDMN broth supplemented with 0.1% sodium taurocholate) for enrichment. The culturing after enrichment involved an ethanol shock for spore selection, followed by plating on CDMN and ChromID agar. Strain isolation was performed through three successive subcultures of single colonies. Bacterial DNA was prepared using the DNeasy Blood and Tissue kit (Qiagen). Isolates were confirmed as *C. difficile* using *cdd3*-PCR (26) and further characterization of *C. difficile* was performed as described (25).

For isolation of *Clostridium* (*C*.) *perfringens*, approx. 0.5 g of feces was resuspended in 10 ml Selzer broth (27) and spread onto Schaedler Agar (PB5034A, Oxoid, Microbiology Thermo Fisher Scientific) and neomycin blood agar (Columbia-Agar, 17.5 g Glucose per L, 10% sheep blood and 100–125 mg/L neomycin) and incubated anaerobically. Colonies suspicious of being *C. perfringens* were subcultured to obtain pure cultures. DNA was prepared from culture material using the DNeasy Blood and Tissue kit (Qiagen) and subjected to PCR as described elsewhere (28).

### 2.10 Screening for *Campylobacter* spp.

Fecal swabs were initially cultured on Campylobacter Blood-Free Selective Medium (CM0739B) supplemented with CCDA Selective Supplement SR0155 (both Oxoid, Thermo Fisher Scientific, Basingstoke, United Kingdom) and incubated at 37 °C for 48 h in an anaerobic jar under a microaerobic atmosphere (5% O_2_, 10% CO_2_ and 85% N_2_) using CampyGen™ (CN0025A, Oxoid). Presumptive single colonies were subcultured on Brain Heart Infusion (BHI) agar (CM1135B, Oxoid) and incubated again for 48 h. All isolates were verified by MALDI-TOF MS (Biotyper microflex LT/SH, library v.10.0.0.0, Bruker Daltonics, Bremen, Germany).

### 2.11 Screening for *Salmonella* spp.

Analysis of fecal samples was carried out according to DIN EN ISO 6579-1. Buffered peptone water (BPW), modified semi-solid Rappaport-Vassiliadis (MSRV), xylose lysine deoxycholate agar (XLD) and Rambach agar (RB) were applied for the analysis (all from sifin diagnostics, Berlin, Germany).

### 2.12 Screening for Shiga toxin-producing *Escherichia coli*

One gram of pooled fecal material was resuspended in 9 ml of BPW (VWR International, Darmstadt, Germany). Aliquots (100 μl) of the supernatant, representing different dilutions, were spread by plating onto Gassner agar plates (sifin diagnostics) and incubated at 37 °C overnight. Plates containing between 300 and approx. 1,000 colonies were washed off with 2 ml LB containing 75% glycerol, an aliquot removed and the remaining bacterial pool suspension was stored at −80 °C. The aliquot from the wash-off was heat-denatured (95 °C; 10 min), briefly centrifuged and stored at −20 °C. The heat-denatured samples of the bacterial pools were used to detect *E. coli* via the *uidA* gene and to screen for the Shiga toxin encoding genes *stx1* and *stx2*. The target sequences were amplified in a multiplex PCR with primers described previously (29, 30) at a concentration of 10 nmol/ml each in a single-tube multiplex PCR using the OneTaq 2X Master Mix with Standard Buffer (New England Biolabs, Frankfurt/Main, Germany) and 2 μl of DNA extract (heat-denatured). Thermocycling conditions were as follows: 94 °C for 30 s, 30 cycles of 94 °C for 30 s, 56 °C for 40 s, and 68 °C for 40 s; and a final extension at 68 °C for 5 min. The PCR-amplified fragments (in a volume of 10 μl) were separated by electrophoresis for 55 min at 100 V in a 1.5% agarose gel stained with Gel red (Merck, Darmstadt, Germany) and visualized under UV light.

### 2.13 Data analysis

As some of the animals were sampled repeatedly, sample positivity rate and animal positivity rate were calculated and are presented herein. Sample positivity rate reflects the number of samples that yielded positive results among all samples tested. In order to calculate the animal positivity rate, those SAC that were tested multiple times were defined as positive if at least one of the samples yielded a positive result. Statistical analysis was conducted using Microsoft Office Excel (Microsoft Corporation, Redmond, WA, United States) (Fisher's Exact test). Confidence intervals were calculated by Microsoft Excel using the Wilson score method and formula CI = ($\hat{\text{p}}$ +Z^2^/2n)/(1+Z^2^/*n*) ± Z/(1+Z^2^/*n*) √[($\hat{\text{p}}$(1-$\hat{\text{p}}$))/*n* + Z^2^/4n^2^]. Figures were built using GraphPad Prism (GraphPad Software, LLC, Boston, MA, United States) (version 10.4.0.621).

## 3 Results

In total, 739 blood and 723 fecal samples were collected from 449 animals. Of these, 292 were alpacas, 156 llamas and one huarizo. Absolute numbers of samples tested may thus vary between tests.

### 3.1 Detection of antibodies against viral pathogens

In the first three sampling rounds, all sera tested negative for antibodies against ruminant pestiviruses. In the 4th round, one sample from an alpaca reacted seropositive (flock 1; sample 1D71), while the remaining samples tested seronegative as in the previous rounds. The positive result was confirmed by means of a neutralization test, the sample reacted positive against BVDV-1 with a titer of 80. Negative results were obtained against BVDV-2 and BDV (titer < 5). In contrast, antibodies against SBV were detected in multiple animals as 54.6% (*n* = 245/449; 95% CI: 49.9%−59.11%) of the sampled camelids tested seropositive in at least one sample. In the first sampling round 48.2% (41.4%−55.1%) of the animals tested seropositive, in the second round 56.7% (49.4%−63.7%), in the third round 54.4% (47.2%−61.6%), and in the final sampling round 50.6% (43.3%−57.8%). In every sampling round, at least one seropositive animal was found in each flock that was included. The proportion of animals with at least one positive sample within the individual flocks ranged from 25.0 to 73.8%.

All serum samples reacted negatively for BoHV-1 antibodies irrespective of sampling round.

**Figure 1:**
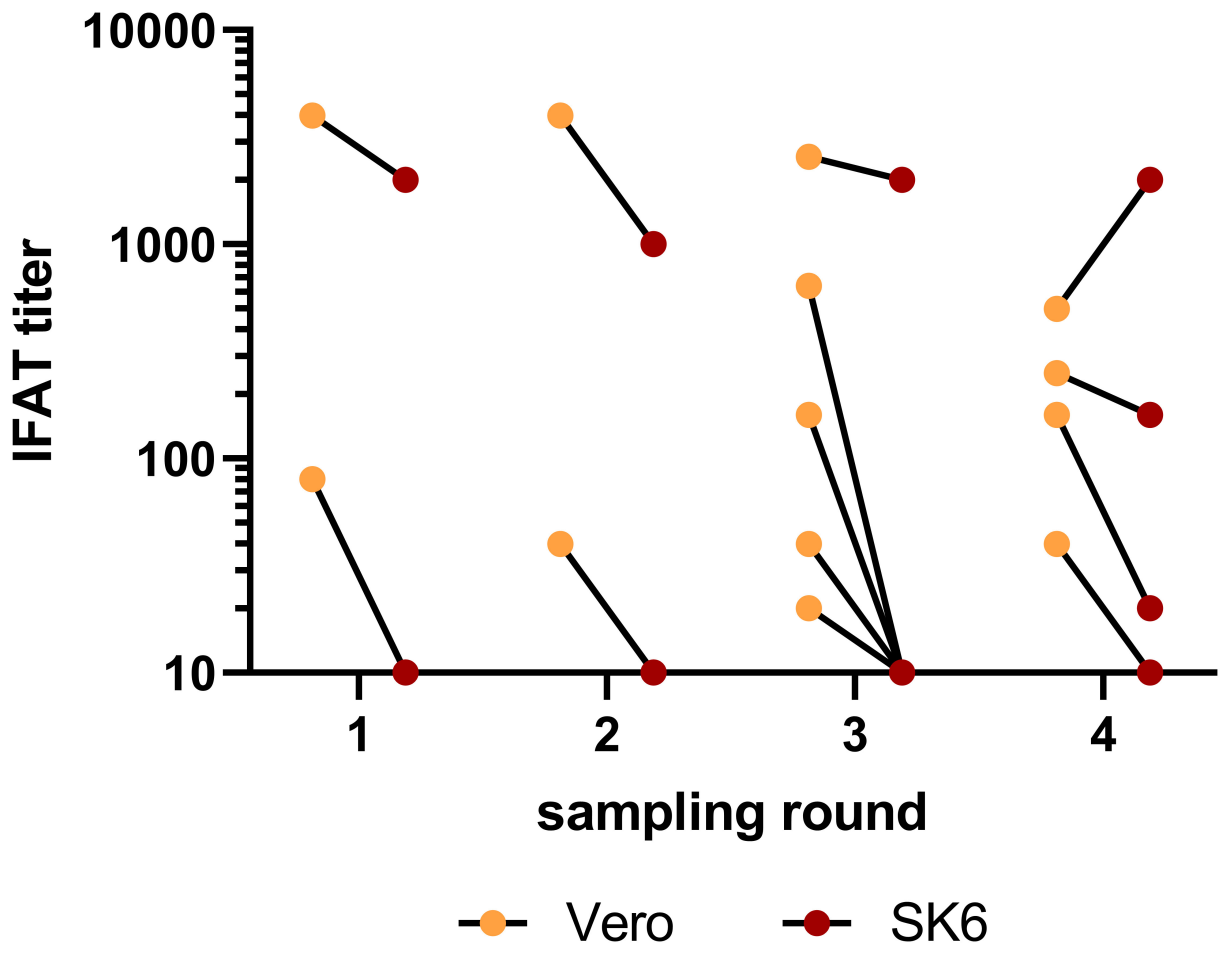
Seroreactivity against BoDV-1 determined by a two-step IFAT screening. Results are presented as IFAT titers in two different variants of the assay using either formalin-fixed Vero cells or heat-fixed SK6 cells. Lines connect results originating from the same sample. Only results of samples with a reactivity in at least one assay are shown.

Thirteen samples originating from nine individuals across six flocks reacted positive in the initial IFAT screening for BoDV-1-reactive antibodies using formalin-fixed Vero cells with titers ranging from 20 to 4,000. Only six of these samples likewise tested positive in the confirmatory IFAT using heat-fixed SK6 cells. Four of these samples originated from a single llama from flock 2, which possessed high titers (500–4,000) in both IFAT variants at all four time points. The remaining two samples originated from a llama and an alpaca from flocks 8 and 10, displaying low to moderate titers (20–250; Figure 1). Overall, 3 out of 449 examined individuals (0.7%) showed reactivity variants against BoDV-1 in both IFAT in at least one sample.

### 3.2 Detection of Candidatus *Mycoplasma haemolamae*

The animal positivity rate for Candidatus *Mycoplasma haemolamae* was 31.6% (*n* = 142/449; 95% CI: 27.5%−36.1%) and varied significantly (*p* < 0.05) between alpacas (28.4%, *n* = 83/292; 95% CI: 23.6%−33.9%) and llamas (37.8%, *n* = 59/156; 95% CI: 30.6%−45.6%). Positive animals were detected in each flock. The proportion of positive animals within a flock ranged from 1 to 100%.

### 3.3 Detection of antibodies against brucellae

Of the 739 samples tested, eight were positive in the iELISA. Two animals were positive at two time points, i.e., a total of six animals were positive in the iELISA at least once. In addition, two samples (from two different animals) were classified as questionable. However, all of these 10 samples gave negative results when tested by CFT.

### 3.4 Indirect and direct evidence for *Coxiella burnetii*-infections

**Figure 2:**
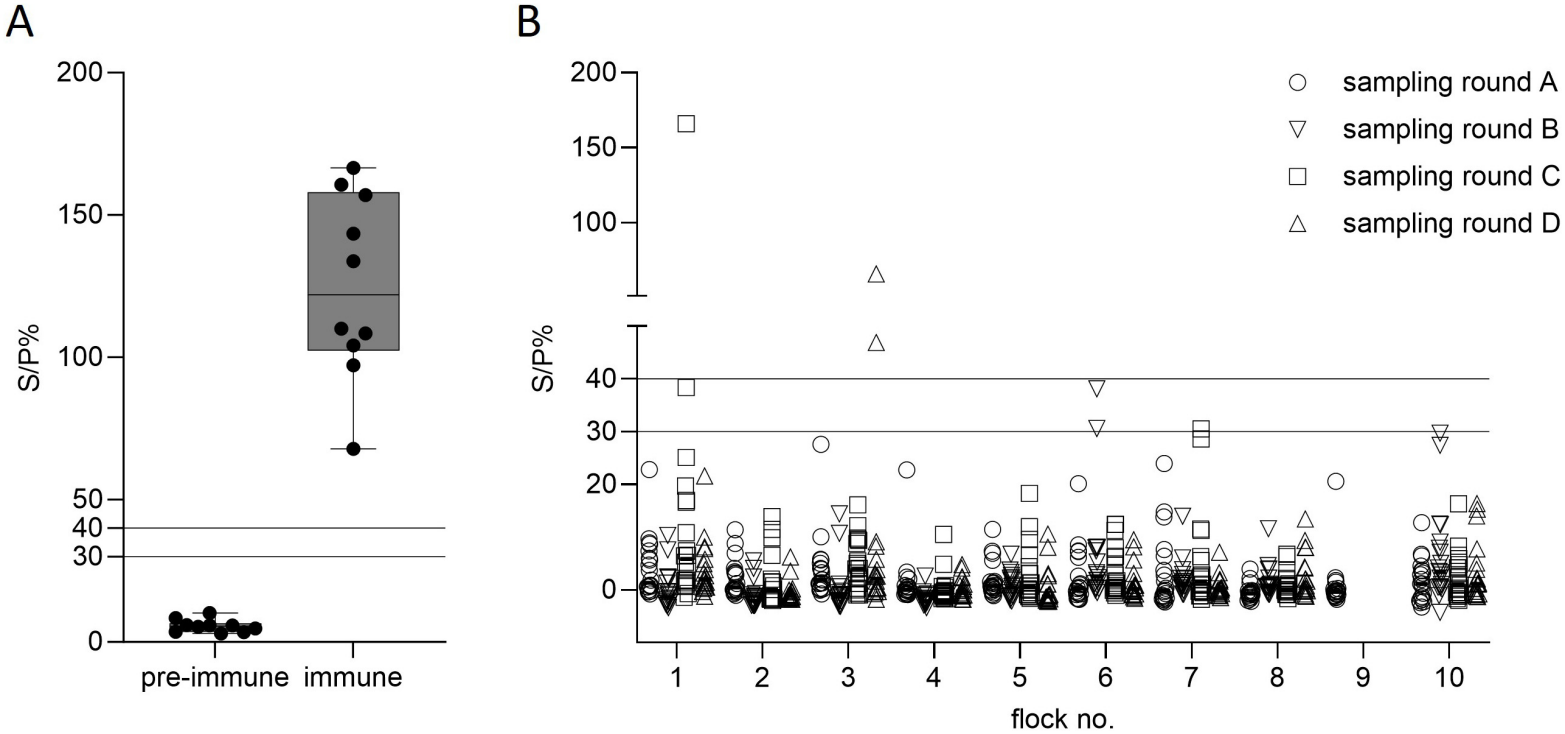
Functionality **(A)** of the IDEXX Q fever (*Coxiella burnetii*) antibody test kit (IDEXX laboratories) and detection **(B)** of *C. burnetii*-reactive samples from South American camelids. Functionality was tested using pre-immune and immune serum from a COXEVAC vaccinated alpaca. Samples were tested five times in duplicate and results illustrated as Tukey box plots. Serum samples (*n* = 738) from 449 animals from 10 different flocks (1–10) were sampled four times (A–D). Solid lines at S/P% 40 and 30 indicate values for positive (>40%), questionable (≤30% to <40%) and negative (<40%) samples.

There is no licensed serological coxiellosis test available for SAC to date. Therefore, the functionality of the ELISA method employed here was demonstrated with pre-immune and immune sera from a vaccinated alpaca (Figure 2A). A pre-immune serum tested negative with a mean S/P value of 5.6 ± 2.2% (Mean ± SD) and an immune serum tested positive with 124.9 ± 32.3%. *C. burnetii* specific antibodies were detected in 3/738 serum samples from three individual animals of two different flocks (Figure 2B). The positive samples were from llamas only belonging to flock 1 (1C60) and flock 3 (3D73, 3D76). The corresponding animals were tested only once (1C60, 3D76) or tested negative in sampling rounds A to C, but positive in round D (3D73). Obtained S/P values were 166.1% (1C60) for the sample from flock 1 or 46.9% (3D73) and 65.7% (3D76) for samples from flock 3. The sample and animal positivity rates were 0.4% (3/738) and 0.7% (3/449), respectively. Questionable results with a S/P value between 30% and 40% were detected in flock 1 (1C47), flock 6 (6B31, 6B35) and flock 7 (7C57) from one llama and three alpacas. These animals were tested only once (1C47), tested negative before (6B31) or tested negative before and/or afterwards (6B35, 7C57).

Fecal samples (*n* = 717) were assessed by IS1111 qPCR. Of all samples, 87 showed inhibitory activity and were excluded. This mainly affected sampling rounds C and D. Of the remaining samples, 1/630 (4A05) tested positive for IS1111 with a Cq value of 34.7. The corresponding alpaca was tested only once. Questionable samples from alpaca (*n* = 4) and llama (*n* = 5) were detected in flocks 4, 5, 6 and 8. Obtained Cq values ranged from 35.1 to 36.8. The positivity rates at the sample and animal levels were determined as 0.2% (1/630) and 0.2% (1/414), respectively. Of note, seropositive animals described in the previous paragraph were negative for IS1111 in fecal samples, throughout.

### 3.5 Indirect and direct evidence for *Chlamydia spp*.-infections

*Chlamydiaceae*-specific antibodies were detected in nine out of 10 flocks, in 13.6% of animals (*n* = 61/449; 95% CI: 10.7%−17.1%) and in 12.7% of serum samples (*n* = 94/739; 95% CI: 10.5%−15.3%). Animal seropositivity rate in individual flocks ranged from 0 (flock 9) to 33.3% (flock 2) and was significantly higher (*p* < 0.01) in llamas (18.1%) than in alpacas (9.1%). There was no significant seasonal variation. The sera did not contain any antibodies specific to *C. abortus* as detected by the IDScreen ELISA.

Chlamydia shedding was detected in eight out of 10 SAC flocks and in 29.6% of animals (*n* = 131/442; 95% CI: 25.6%−34.1%) with a sample positivity rate of 20.9% (*n* = 150/719; 95% CI: 18.1%−24.0%). Values were similar (*p* > 0.05) in llamas and alpacas with animal positivity rates of 28.5% (*n* = 43/151; 95% CI 21.9%−36.1%) and 30.0% (*n* = 87/290; 95% CI: 25.0%−35.5%), respectively. The rate of *Chlamydiaceae*-positive animals within the individual flocks ranged from 0% (flock 5 and 9) to 53.8% (flock 10). Intermittent shedding was observed with higher shedding rates in winter (sampling rounds B and D with a sample positivity rate of 28.7%) compared to summer (sampling rounds A and C with a sample positivity rate of 13.5%; *p* < 0.01). Low chlamydial loads, represented by Cq values of 35–38, were detected in the majority of positive samples (49.7%), while only 6.1% of positive samples had a Cq value < 25, indicating high chlamydial loads.

Typing by species-specific PCR identified a *C. suis*-like bacterium as the predominant species in 74.1% (*n* = 97/131; 95% CI: 66.5%−81.6%) of the *Chlamydiaceae*-positive animals, followed by *C. pecorum* in 20.6% (*n* = 27/131; 95% CI: 14.6%−28.3%) of these animals. Interestingly, *C. abortus*, an abortifacient in small ruminants, was detected in eleven animals from flock 1 exclusively in sampling round D. The zoonotic *C. psittaci* was not found. However, 10.7% of the *Chlamydiacea*e-positive samples could not be typed, probably due to low DNA content.

### 3.6 Detection of antibodies against *Mycobacterium tuberculosis* complex (MTC)

**Figure 3:**
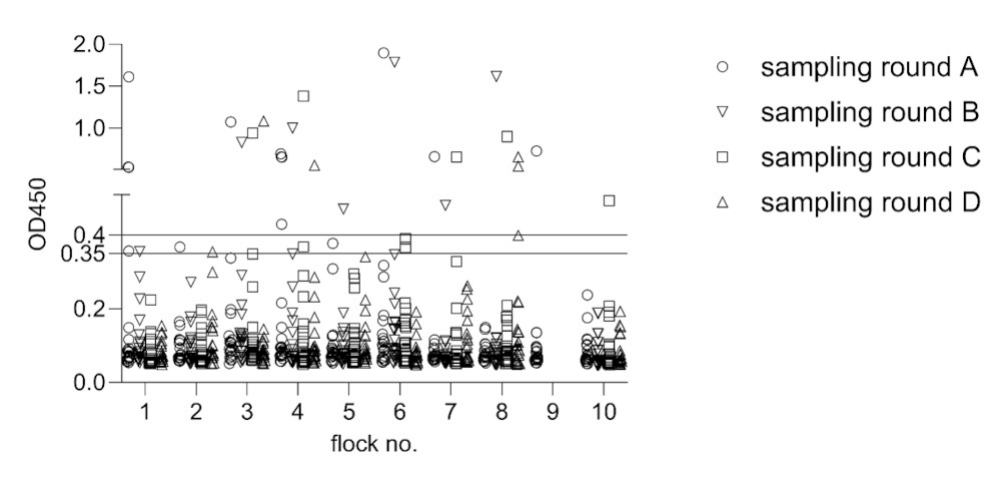
Detection of tuberculosis specific antibodies in serum samples of South American camelids using the Tuberculosis DR-ELISA. Values (mean of duplicates) are depicted according to the flocks (1–10) and sampling round (A–D). Solid lines at OD_450*nm*_ 0.4 and 0.35 indicate values for positive (>0.4), questionable (≤0.35 to <0.4) and negative (<0.35) samples.

Of the 739 serum samples, 703 (95.1%) were assessed as negative in the Tuberculosis DR-ELISA while 36 (4.9%) were non-negative (26 as positive and 10 as questionable). Non-negative sera were determined in all 10 farms examined (Figure 3). No seasonal pattern was observed between summer (samplings A and C) and winter (samplings B and D). When assigning non-negative samples to individual animals, most detections, namely 27, occurred only once per animal, even though additional serum samples were tested from 16 of these animals. In only three animals, two-, three or four-fold detection of non-negative samples was achieved. No significant difference was observed between the host species, as 12 of 156 llamas (7.7%) and 18 of 292 (6.2%) alpacas tested non-negative (*p* > 0.05; data not shown).

### 3.7 Detection of infections with *Mycobacterium avium* subsp. *paratuberculosis* (MAP) and other non-tuberculous mycobacteria

In total, 376 fecal samples from 295 animals were analyzable by culture. MAP was isolated from one of three fecal samples obtained from one llama in flock 5. No other fecal sample from that flock and from all other flocks was culture positive. Accordingly, MAP was detected in 0.3% of the samples and 0.34% of the animals examined. Other non-tuberculous mycobacteria [3 × *Mycobacterium* (*M*.) *shimoidei*, 1 × *M. setense*] were identified in the fecal samples of the first sampling round in flock 7 while the second sampling round yielded negative results throughout.

### 3.8 Detection of *Clostridioides difficile* and *Clostridium perfringens*

In total, 370 samples (*n* = 194 and *n* = 176 samples in sampling rounds A and B, respectively) from 293 animals (with 77 animals sampled twice), were tested for the presence of *C. difficile* and *C. perfringens* by bacterial culture. Samples were derived from alpacas (*n* = 230 samples obtained from 195 individual animals) and llamas (*n* = 139; 97 animals), and one huarizo.

In the first sampling round (A), *C. difficile* was isolated from two fecal samples from flock 4. Both isolates were obtained by enrichment culture and possessed the toxin genes for toxin A and toxin B, consistent with isolates typically associated with *C. difficile* infections (CDI). One isolate was assigned to ribotype (RT) 020 and the other to RT 076. In the second sampling round (B), *C. difficile* was isolated from six fecal samples from four flocks (3, 4, 6, 10). All isolates were obtained by enrichment culture and harbored the toxin genes for toxin A and toxin B except for one isolate lacking toxin genes. The isolates were assigned to different ribotypes. One sample (4B38) from flock 4 tested positive for *C. difficile* and *C. perfringens*. Overall, the *C. difficile* sample positivity rate was 2.2% (*n* = 8/370; 95% CI: 1.1%−4.2%) and the animal positivity rate 2.7% (*n* = 8/293; 95% CI: 1.4%−5.3%). All positive animals were alpacas, corresponding to a sample positivity rate of 3.5% (*n* = 8/230; 95% CI: 1.8%−6.7%) and an animal positivity rate of 4.1% (*n* = 8/195; 95% CI: 2.1%−7.9%).

In round A, 21 *C. perfringens* isolates were obtained from 8 of 10 farms, 12 *C. perfringens* isolates from 5 of 9 farms in round B. Positive fecal samples contained low *C. perfringens* bacterial counts. All isolates were identified as *C. perfringens* type A with two isolates carrying the gene for the beta 2 toxin. The overall sample and animal positivity rates were 8.9% (*n* = 33/370; 95% CI 6.4%−12.3%) and 10.9% (*n* = 32/293; 95% CI: 7.8%−15.0%), respectively, including one repeatedly positive animal. For llamas the sample positivity rate was 8.6% (*n* = 12/139; 95% CI: 4.0–13.3) and the animal positivity rate was 12.4% (*n* = 12/97; 95% CI: 5.0%−14.5%). For alpacas, the sample positivity rate was 9.1% (*n* = 21/230; 95% CI: 6.0%−13.6%) and the animal positivity rate was 10.8% (*n* = 21/195; 95% CI: 7.2%−15.9%).

### 3.9 Detection of *Campylobacter* spp.

A total of 717 fecal swabs, collected from 286 alpacas (427 samples), 152 llamas (288 samples), and one huarizo (2 samples), were analyzed. Thermotolerant *Campylobacter* isolates (16 *C. jejuni* and 7 *C. coli*) were cultured from 23 swabs (3.2%; 95% CI: 2.1%−4.8%) originating from 21 animals (4.8%; 95% CI: 3.2%−7.2%). Two animals tested positive at two different sampling time-points. Most isolates were obtained from flock 4, where both species were detected (*C. jejuni*: eight isolates; *C. coli*: six isolates), resulting in an intra-flock positivity rate of 17.5% (95% CI: 10.7%−27.3%) at the sample level and 23.2% (95% CI: 14.1%−35.8%) at the animal level. There was a clear and statistically significant seasonal variation (*p* < 0.05). During the summer months, the sample positivity rate was 4.6% (95% CI: 2.9%−7.2%), which was higher than the 1.7% observed during the winter months (95% CI: 0.8%−3.7%). Alpacas were significantly (*p* < 0.01) more often affected than llamas, with animal positivity rates of 6.6% (95% CI: 4.3%−10.1%) and 1.3% (95% CI: 0.4%−4.7%), and sample positivity rates of 4.9% (95% CI: 3.2%−7.4%) and 0.7% (95% CI: 0.2%−2.5%), respectively.

### 3.10 Detection of *Salmonella* spp.

*Salmonella* organisms were found in only 1 of the 719 fecal samples examined. *Salmonella* (*S*.) Typhimurium was detected in an alpaca in flock 6 (6D63). This animal had already been tested with a negative result (6C50) approximately 7 months earlier.

### 3.11 Detection of Shiga toxin-producing *Escherichia coli*

A total of 716 fecal samples from 442 animals were tested for the presence of genes encoding *stx1* and *stx2*. The overall rate of *stx*-positive samples across all 10 flocks and four sampling rounds was 32.4% (232/716; 95% CI: 29.1%−35.9%). This resolved into positivity rates of 5.0% (36/716; 95% CI: 3.7%−6.9%), 19.3% (138/716; 95% CI: 16.6%−22.3%) and 8.1% (58/716; 95% CI: 6.3%−10.3%) for *stx1*-, *stx2*- and *stx1*/*stx2*-positive samples, respectively. The corresponding animal positivity rate for the presence of an *stx*-gene was higher with 42.3% (187/442; 95% CI: 37.8%−47.0%). The rate was quite diverse for the individual farms with values between 10% (2/20) to 62% (23/37). We did not detect a difference in animal positivity rates between alpacas (43.1% 125/290; 95% CI: 37.5%−48.9%) and llamas (41.1%; 62/151; 95% CI: 33.5%−49.0%). The sole huarizo, which was tested only once, was *stx*-negative. The intra-flock *stx*-sample positivity rate ranged widely from 0% (0/20) to 95% (19/20) across all sampling rounds, resulting in a median value of 35.0% (95% CI: 33.3%−48.9%). During the first sampling round, nine of 10 flocks were *stx* PCR-positive. In the remaining three sampling rounds, all nine farms visited were PCR-positive for *stx*.

## 4 Discussion

Accounting for the growing numbers and intensified use of SAC in Europe, and in Germany in particular, a project was initiated in cooperation with one of the German breeder associations (Verein der Züchter, Halter und Freunde von Neuweltkameliden e.V.). Its overarching aim was to establish an animal health management plan to improve animal health and welfare on SAC-holding farms. To this end, one objective was to generate data on the occurrence of a range of viral and bacterial pathogens of prime importance.

Although only 10 holdings were visited for this study, they were distributed throughout the country and selected to mirror the diversity of German SAC farms in size and use of the animals. For welfare reasons and to gain unbiased occurrence data for the regular population, only animals without obvious clinical disease were sampled in the flock, i.e., in the immediate environment they are accustomed to. This approach may have led to underestimating prevalence data, at least for pathogens known to be shed in more abundant numbers under stress-full conditions (31), e.g., when animals are handled by persons they are not used to on the occasion of trekking events or animal-assisted therapy, or if the animals are undernourished (32). Exclusive sampling of non-diseased animals also limits conclusions regarding the pathogenicity of some agents for SAC, for which no correlation with clinical disease in these animals has yet been established (e.g., *C. difficile*).

Diagnostic methods applied herein followed WOAH or EU-RL recommendations, whenever available, although not all are validated for use in SAC. Where feasible, cultural and PCR-based methods were deployed according to established workflows to apply the most reliable (in case of positive results) and sensitive method for the agent in question. Serological tests, often used to detect systemic infectious with no or undetectable shedding, are prone to false-positive and -negative results. This particularly applies to sera from camels and SAC as they produce two types of immunoglobulin γ (IgG), i.e., the mammalian IgG and structurally smaller so-called heavy chain IgG antibodies (HCAb). The latter reportedly can represent 75% of the total IgG content in serum (33–35), which may influence the functionality of test systems used and lead to erroneous results in indirect tests. As discussed in detail below, low numbers of positive test results from serological assays reported herein may represent either false-positives or underestimations whereas high numbers may indicate overestimations. It should also be noted, that estimated occurrence data deduced from direct and indirect detection methods in this study are difficult to compare across the pathogens investigated.

While control programs for BoHV-1 and BVDV have been in place in bovines for more than a decade, leading to an official disease-free status for the entire country (BoHV-1) or the most of its area (BVDV) (36), SBV has established an enzootic status in the ruminant population since its first appearance in 2011 (37). Accordingly, BoHV-1 and BVDV antibodies were either absent or detected in only a single SAC animal in this study, whereas antibodies against SBV were detectable in every flock. These findings suggest that SAC have no decisive relevance for the BoHV-1 control program in Germany, which aligns with unpublished serological results for more than 1,500 samples collected in a comprehensive study in 2008/2009 (38). For SBV, on the opposite, the results imply that SAC have become an integral part of the natural SBV transmission cycle between its insect vector *Culicoides* biting midges, and the mammalian hosts in the country (39). In a previous experimental infection study, no obvious clinical signs or gross pathological lesions were observed in any of the infected llamas or alpacas, though a short-lived viremia followed by seroconversion was induced (39). Similarly, participants in a field study conducted in Germany from September 2012 to December 2013 reported no clinical signs or increase in abortion or congenital malformation associated with fetal SBV infection (39). Therefore, it is assumed that SBV causes only subclinical infection in SAC.

BoDV-1 is transmitted by its natural reservoir, the bicolored white-toothed shrew (*Crocidura leucodon*), and present in their populations in a restricted endemic area covering only parts of Germany, Austria, Switzerland and Liechtenstein (40). Sporadic spill-over transmissions to a broad range of other mammals, particularly horses, sheep, SAC and humans, cause severe and usually fatal non-suppurative encephalitis. In these spill-over hosts, the virus is strictly neurotropic and, hence, they act as dead-end hosts (12, 41). In this study, only 0.7% of the animals showed positive reactivity in both BoDV-1 IFAT variants in at least one tested sample and only a single individual exhibited consistently high titers across all samples, demonstrating the low seroprevalence in SAC not showing signs of Borna disease both inside and outside the known endemic area. The llama with the consistently high BoDV-1-reactive titers originated from a region in Hesse, which is currently not recognized as BoDV-1-endemic (40). However, since unspecific reactivities are not uncommon in bornavirus serology, direct virus detection from the brain would be required to confirm BoDV-1-specific seroconversion (15, 41–43).

A conventional PCR was deployed for the detection of *Mycoplasma haemolamae* which may yield false-negative results. However, this method is the most commonly used in previous studies and currently represents the only approach allowing sensitive and specific detection of the agent (44, 45). Indeed, the individual animal positivity rate of *M. haemolamae* found in this study is at a similar level as reported for other European countries, including Austria (25.8%), England (29.0%) and Switzerland (18.0%) (45–47) as well as for the U.S. (9.2 %) and Peru (18.4%) (48, 49), implying that the pathogen is widespread in the global SAC population.

Brucella infections are common in Old World camels but rare in SAC (50). However, serological monitoring implies that brucellosis also occurs in alpacas (51), and infections in llamas have been induced experimentally (52). Accordingly, an infection with *B. melitensis, B. abortus* or *B. suis* in camels is classified as category D (prevent disease from spreading) and E (surveillance requested) diseases under Commission Implementing Regulation (EU) 2018/1882. In this study, antibody testing followed the German brucellosis monitoring scheme for cattle, using ELISA followed by CFT as a confirmatory test. Ten out of 449 samples (approx. 2.2%), yielded positive or questionable ELISA results (presumptive false-positives), which all reacted negatively in the subsequent CFT and therefore did not meet the legally binding case definition under EU law (Commission Implementing Regulation (EU) 2020/689). While the cut-off values used are validated for cattle, but not for camelids, the observed number of 2.2% non-negative ELISA rate is consistent with the expected diagnostic specificity for bovines (53, 54). False-positive serological reactions are a well-known issue in brucellosis surveillance (55), especially in countries like Germany with an officially brucellosis-free status in cattle, sheep and goats (Decision 99/466/EC and 93/52/EEC).

North African camelids are considered susceptible to several zoonotic abortifacient pathogens including *C. burnetii* (56), for which seroprevalence values exceeding 50% have been reported (57, 58). Excretion of *C. burnetii* in camel milk has been detected by PCR, and associations between positive serology and abortions have been established in a few cases (59, 60). The prevalence and pathogenesis of coxiellosis in SAC, however, remain vastly unknown. Our study allows to estimate the positivity rate as 20% and 10% at the flock level using indirect (ELISA) and direct methods (qPCR), respectively, and less than 1% for both approaches at the animal or sample levels. Similarly, 619 sera from 536 animals in Austria were serologically tested for coxiellosis, with only two samples evaluated as positive or questionable (61). However, the here presented data suggest that the applied ELISA method is applicable to camelids, but cut off values for positive, negative or doubtful results need validation. *C. burnetii* has been detected in two abortions in Belgium, and in five abortions in Switzerland, partly as a member of a co-infection (62, 63). In the present study, most fecal samples were negative, a few were classified as questionable and only one sample tested positive, albeit with a high Cq value. It remains unclear whether *C. burnetii* is excreted in feces of SAC during infection. To clarify the role of SAC in the epidemiology of coxiellosis, applied tests and additional matrices, e.g., vaginal swabs shortly after foaling or milk, should be validated and analyzed, and animals should be monitored more closely.

Chlamydiae were included in this study because of their potential role as pathogens and zoonotic agents (64). In eight out of 10 SAC farms and in 29.4% of the animals, fecal shedding of chlamydia was detected, and 13.6% of the animals tested positive for chlamydia-specific antibodies. These findings suggest, that intestinal carriage of chlamydiae is endemic in German SAC, with a prevalence potentially higher than reported in a previous study using pooled fecal samples from 43 farms, which found a sample prevalence of 7.4% (95% CI 2.1%−12.7%) and a flock prevalence of 14.0% (95% CI 3.6%−24.3%) (8). The predominant chlamydial agent identified is a novel *C. suis-*like *Chlamydia* species whose phylogeny and pathogenic potential require further investigation. No clinical symptoms were observed in SAC shedding this agent or other chlamydiae, such as *C. pecorum* or *C. abortus*. The latter is a known abortifacient in sheep (65) and is suspected of causing reproductive disorders in SAC, although scientific evidence is limited (56, 66). Its sporadic detection in flock 1 in only one sampling round may be interpreted as environmental contamination from ruminant shedders on the same farm rather than an establishment in the SAC flock. This assumption is further substantiated by the negative serological findings of *C. abortus* antibodies in these animals. To further evaluate the role of *C. abortus* in SAC, vaginal, fetal or placental samples should be examined when reproductive failures occur. While chlamydiae are generally suspected of negatively affecting fertility and of being zoonotic, however, a positive chlamydia test should be considered only a minor risk as long as abortifacient *C. abortus* or zoonotic *C. psittaci* are not detected, emphasizing the need for differential diagnosis at the chlamydial species level.

Animal tuberculosis (aTB), caused by members of the MTBC, is a zoonotic disease with global distribution. While cattle infections attract the most attention due to their high zoonotic impact, infections have been reported in nearly all mammalians and birds. This broad host range is reflected in Regulation (EU) 2018/1882 that categorizes MTBC infections in cattle as category B (eradication), in artiodactyla as category D (prevent disease from spreading), and in mammalia as category E (surveillance requested) disease. For SAC, the categorization as D disease resulted in a mandatory pre-movement surveillance program including, beside other requirements, the obligation to test breeding animals annually for aTB with negative result (regulation (EU) 2020/688). The European Reference Laboratory for Bovine Tuberculosis (EU-RL bTB) lists the tuberculin skin test (SOP/003/EURL) and one ELISA test (SOP/005/EURL) as recommended diagnostic tools for surveillance of aTB in camelids (www.bovinetuberculosis.eu). Tuberculosis in alpacas has been a reportable disease in Germany since 2005. Between 2007 and 2019, only six cases of tuberculosis in camelids were listed in the national animal disease reporting system (TSN), thereof three caused by *M. avium* subsp., two by *M. microti*, and one by an unspecified mycobacterial species. This indicates a very low prevalence, if any at all, of aTB in German SAC. Across Europe, aTB in SAC has been reported in several countries, i.e., the United Kingdom (67), Ireland (68), Poland (69), Spain (70), and the Netherlands (71). Notably, cases in Poland and the Netherlands occurred due to import of aTB-positive animals highlighting the importance of trustworthy pre-movement testing to protect TB-free populations. A recent review of the cause of SAC mortality in Ireland identified *M. bovis* in 27.4% of the submissions and TB as the leading pathology detected in adult alpacas examined (3). In the present study, the ELISA recommended in the SOP of the EU-RL for bTB identified 4.9% of samples and 6.0% of SAC as non-negative. All flocks included in the study can be considered aTB-free based on history, clinical status of the animals, and epidemiological investigations. Moreover, the proportion of non-negative results is consistent with specificity values reported for this ELISA (72), despite the fact that animals were not primed with a tuberculin skin test 15–30 days prior to serum collection as recommended by the EU-RL. Despite this, a test with this specificity value is not applicable in an aTB-free population as it would lead to an inacceptable high number of (presumptive false) positive results. This is supported by the observation that only for 3 of the 19 non-negatively tested SAC, for which more than one serum sample was available, the non-negative result could be reproduced. Our data suggest that the test specificity must be increased for broad field application, either by modifying the ELISA protocol [e.g., using higher serum dilutions or adjusting the cut-off as suggested by Bezos et al. (72)] or by using additional serological tests as suggested by Rhodes et al. (67).

Only limited data are available on the prevalence of MAP infections in SAC, both in their natural habitats in the Andes of South America and in the countries to which they were exported to (8). Yet, SAC are susceptible to MAP and individual cases have been reported in German zoos and private holdings (73, 74). Conducting large scale prevalence studies is hampered by the lack of validated ELISA tests for the detection of MAP antibodies in SAC sera. For this reason, MAP prevalence was assessed by direct methods in this study. The agent was isolated from 0.27% and 0.34% of the samples and the animals, respectively, pointing to a very low prevalence in the 10 flocks included. The presence of genome fragments of MAP could not be excluded in one 5-sample pool from flock 4 (Cq value 38.8), but attempts to culture MAP from individual fecal samples failed. An estimated very low prevalence of MAP is corroborating recent results from 43 farms and zoos in Central Germany, with negative results throughout (8). Studies from the Tierra del Fuego Island and from the Chilean Altiplano indicate that the prevalence of MAP infection is also rather low in wild and domestic SAC in their countries of origin (75). However, there remains a risk of infection if these animals are kept in or moved to regions where paratuberculosis is endemic. For example, transmission of MAP between domestic sheep and wild guanacos grazing on the same pasture has been reported (76). Notably, the MAP genotypes isolated from SAC in Germany were identical or similar to genotypes found in domestic cattle, goats and wild ruminants, indicating potential interspecies transmission (73).

SAC must be considered a potential reservoir for human pathogenic *C. difficile*, as the ribotypes identified in this study have also been detected in human infections. However, the number of carrier animals was low, as previously reported by Dost et al. (25). *C. perfringens* was obtained from animals without documented gastrointestinal tract (GIT) disease during this study, yet is widespread in the GIT of many animal species as well as in the environment (77). The isolates, which were recovered in only low bacterial numbers per sample, are therefore likely to represent part of the normal intestinal microbiota of SAC, even though the beta 2 toxin is considered a potential virulence factor (78).

Thermotolerant *Campylobacter spp*. have not previously been reported in SAC, but were detected on the majority of German farms sampled, with *C. jejuni* being more frequent and widespread (16 isolates from seven farms) compared to *C. coli* (seven isolates from two farms). Detection of *Campylobacter* was significantly less frequent in llamas, with only two isolates identified on the farm housing llamas exclusively. However, definitive conclusions about differences in host suitability remain difficult as *Campylobacter* spp. were also rare in alpacas (only one *C. jejuni* isolate) on the four remaining mixed farms with both llamas and alpacas. The proportion of positive samples was higher in summer (4.6%) than in winter (1.7%), likely reflecting seasonal effects related to temperature and rainfall, as has been reported for other host species across Europe (79). The repeated detection of clonal *Campylobacter* spp. isolates (confirmed by whole genome sequencing, data not shown) in consecutive samples from a farm or an animal suggests endemic and/or persistent colonization. As observed in other hosts (80), these zoonotic pathogens appear to be part of the microbiota of healthy SAC.

Compared to ruminants, data on the significance and prevalence of *Salmonella* spp. in SAC are scarce (81, 82). Reports of 23 infections caused by seven *Salmonella* serovars, including two abortions associated with *S*. Dublin and *S*. Typhimurium between 2000 and 2015 in England and Wales (4, 83) indicate both, the general occurrence of different *Salmonella* serovars in SAC–with or without clinical manifestation—and a rather low prevalence of only one to two *Salmonella* cases per year in these species. Examination of 719 fecal samples in this study led to the detection of only a single *S*. Typhimurium strain. As this animal had tested negative several months earlier, intermittent shedding appears likely. Overall, the prevalence of *Salmonella* organisms in German SAC appears to be very low corroborating previous findings in which no *Salmonella* organisms were detected in 43 SAC holdings (8). Although SAC farms from only a limited number of German federal states were included and differences in husbandry practices may exist in other regions, the potential zoonotic risk posed by SAC-derived *Salmonella* seems to be low. Nevertheless, given the general infectivity of *Salmonella* organisms for SAC, close contact to other livestock (e.g., sheep, cattle, goats) or shared pastures may serve as a potential source of infection and must be considered for differential diagnosis.

The major STEC reservoir is the ruminant GIT (84), but STEC are also shed by many other animal species (85) including SAC, e.g., in Germany, Peru and the United Kingdom (4, 86–89). The intra-flock positivity rates determined here are similar to ranges published for cattle herds in several studies from Michigan, USA [8.2%−53.7%; (90)], Ireland [52% and 76% across two sampling periods; (91)] and Germany (0%−100%) (92). In two studies differentiating between *stx1* and *stx2* gene presence, the *stx2* gene was detected more frequently than the *stx1* gene, consistent with the findings here. Comparable patterns have also been reported for sheep [as reviewed in McCarthy et al. (93)] and goats (94). In camels, analyses of fecal matter yielded prevalence values of 50% (9/18) in Qatar (95) and 2.7% (1/36) or 5.6% (2/36) for *stx1* or *stx2* gene presence, respectively, in Tunisia (96). Colonization of SAC with STEC thus appears to vary widely between individual farms and to occur at levels similar to those in known ruminant reservoirs. No differences were observed in STEC colonization between alpacas and llamas, nor any seasonality in *stx*-gene presence, in contrast to published data for cattle (97) and pigs (98). Animals sampled repeatedly showed no consistent pattern. Some remained negative, others became positive or lost the *stx* signal with varying *stx* gene profiles–indicative of intermittent STEC shedding as commonly observed for ruminants. Even though the European Food Safety Authority (EFSA) (99) classifies all STEC strains as potential human pathogens, a thorough assessment of the zoonotic risk posed by STEC from SAC requires further characterization of the *stx* gene subtypes and further associated virulence factors. The information on the presence of *stx1* and/or *stx2* alleles in the individual animal samples generated in this study so far is clearly limited in allowing a critical assessment of the public health risk emanating from SAC. It will best be addressed by isolating STEC strains from positive samples and characterizing their virulence factor profiles.

This study differs from the precursor study by González–Santamarina et al. (8) by incorporating a strong longitudinal component rather than being purely cross-sectional, and it sampled individual animals instead of taking composite fecal samples. This might explain the higher proportion of positive farms reported here, as on several farms only a few animals tested positive, and their signal might have been missed in composite fecal samples from the respective farms in the previous study. In order to shed light on the true prevalence of epizootic and zoonotic viral and bacterial pathogens on German SAC farms–at flock level, at the individual animal level, and over time for intermittently shed agents, like MAP, STEC or *Salmonella*–this study serves as a valuable complement to the few cross-sectional studies conducted in Europe to date and helps to better assess the risk posed by SAC to veterinary and public health.

The potential roles of several agents, particularly BoDV-1, *C. burnetii*, chlamydiae, *C. perfringens* and *C. difficile*, as pathogens in SAC awaits to be assessed by analyzing samples from diseased animals. However, evidence is accumulating that, for a number of pathogens, SAC may act as important intermediate hosts in multi-species transmission cycles, most importantly when introduced in increasing numbers into a geographic area where they are non-indigenous. Beyond their potential role as reservoir hosts for epizootic agents, which can pose a risk to livestock health, the detection of foodborne and abortifacient zoonotic agents underscores the importance of maintaining strict hygienic standards. This particularly applies when people interact with these animals, e.g., when SAC are used in animal-supported therapy or tourist activities. Unfortunately, while hygienic guidelines exist for dogs or, more general, for “animals” being deployed in animal-assisted therapy [e.g., (100–102)], no specific guidelines have been established or made publicly available for SAC in Germany thus far.

Measures to monitor freedom from disease and to prevent the spread of animal disease and zoonotic pathogens have long been established in traditionally farmed animals in Europe. The findings presented herein are the result of the first comprehensive study on the occurrence of viral and bacterial pathogens in SAC in Germany and substantiate the concern that SAC can carry various animal disease and zoonotic pathogens. Compared to contemporary data on the prevalence of these infectious agents in livestock species in Europe (https://www.woah.org/en/what-we-do/animal-health-and-welfare/disease-data-collection/) and appreciating the occurrence values and strain characteristics collated during this study, SAC do not appear to pose a greater direct risk than other livestock species. However, since SAC are often kept together with other susceptible animal species, such as cattle or sheep, they are of particular epidemiological importance as a potential source of potential pathogens.

## Data Availability

The dataset for this study is available in the EU Open Research Repository Zenodo, https://zenodo.org/records/17959907.
